# Ketone body supplementation in keto‐adapted mice reveals metabolic adaptations and glycogen‐independent exercise capacity

**DOI:** 10.14814/phy2.70583

**Published:** 2025-09-25

**Authors:** Sihui Ma, Cong Wu, Yishan Tong, Yumiko Takahashi, Katsuhiko Suzuki, Taichi Hara

**Affiliations:** ^1^ Faculty of Sport Sciences Waseda University Tokorozawa Saitama Japan; ^2^ Graduate School of Sport Sciences Waseda University Tokorozawa Saitama Japan; ^3^ Department of Sports Sciences The University of Tokyo Meguro‐ku Tokyo Japan; ^4^ Faculty of Human Sciences Waseda University Tokorozawa Saitama Japan

**Keywords:** AMPK signaling, glycogen sparing, keto‐adaptation, ketone body, metabolic flexibility

## Abstract

Ketone body supplementation has gained attention for its metabolic effects, but its impact on exercise metabolism remains controversial. We hypothesized that the metabolic response to ketone supplementation differs between keto‐adapted and keto‐naïve states. In this study, we investigated the effects of β‐hydroxybutyrate (BHB) supplementation in keto‐adapted mice. Mice were assigned to three groups: control diet (CON), ketogenic diet (KD), or KD with sodium β‐hydroxybutyrate supplementation (KD+BHB) for 6 weeks. Chronic BHB supplementation in keto‐adapted mice (KD+BHB) further elevated circulating ketone levels compared to KD alone (2.63 ± 0.53 vs. 1.96 ± 0.34 mM, *p* < 0.05). Despite significantly lower muscle glycogen content, both KD and KD+BHB groups maintained exercise capacity comparable to controls, demonstrating a glycogen‐thrifty effect. During exercise, both KD groups showed greater BHB utilization and glucose preservation compared to controls. Gene expression analysis revealed upregulation of fatty acid oxidation‐related genes across multiple tissues in KD+BHB mice, with more pronounced effects than KD alone. Additionally, KD+BHB mice showed increased AMPK phosphorylation (*p* < 0.05 vs. CON) and reduced mTOR activation (*p* = 0.058 vs. CON) in liver and skeletal muscle, creating a metabolic environment favoring fat utilization. These findings demonstrate that ketone supplementation in keto‐adapted status creates a glycogen‐thrifty state during exercise, suggesting metabolic context significantly influences responses to exogenous ketones.

## Graphical abstract

Chronic β‐hydroxybutyrate (BHB) supplementation in keto‐adapted mice may further enhance lipid metabolism and suggests a potential glycogen‐sparing effect during exercise, as indicated by gene expression and metabolic profiles.

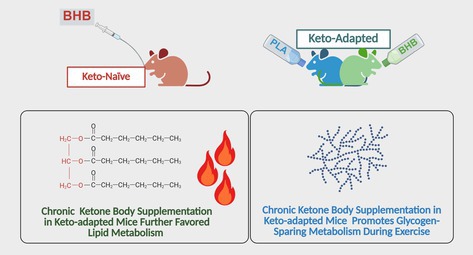

## 1 INTRODUCTION

A ketogenic diet (KD) induces a unique metabolic status called nutritional ketosis through strict limitation of carbohydrate intake. In this state, the concentration of ketone bodies produced by the liver in the body significantly increases, becoming an important energy source for extrahepatic tissues while also exerting signaling roles, such as serving as metabolic regulatory signals released in response to fasting (Wheless, 2008). Prolonged KDs (typically over 2–4 weeks) promote the body's adaptation to utilize fatty acids and ketone bodies as primary fuels, a process termed keto‐adaptation (Sherrier & Li, 2019). In the keto‐adapted state, the body's ability to utilize glucose decreases due to limited glucose availability, while fat mobilization and fatty acid oxidation capacity increase, which potentially favors exercise performance (Ma & Suzuki, 2019; McSwiney et al., 2018). However, keto‐adaptation induced by a KD requires a relatively long time (usually 2–4 weeks in humans) (Wheless, 2008). Therefore, understanding the effects of ketone supplementation in different metabolic states, particularly in keto‐adapted versus keto‐naïve conditions, is of great interest. We operationally define keto‐adaptation as the metabolic state achieved after prolonged (>2 weeks in mice, >3–4 weeks in humans) adherence to a ketogenic diet, characterized by: (1) sustained elevation of blood ketones >1.0 mM in mice (>0.5 mM in humans), (2) upregulation of ketolytic enzymes (BDH1 and OXCT1), (3) enhanced capacity for fat oxidation, and (4) maintenance of physical capacity despite substantially reduced glycogen stores.

Exogenous ketone supplementation has emerged as a promising approach to rapidly achieve nutritional ketosis, potentially broadening its applications and favorable effects on metabolism and exercise performance (Evans et al., 2022; Poff et al., 2020). Exogenous ketone supplementation may provide a means to elevate blood ketone levels without the need for strict dietary restrictions, potentially offering an alternative approach to study the metabolic effects of ketosis. However, it is crucial to note that the ketosis induced by a KD, or an exogenous ketone supplementation, represents two distinct physiological states (Shaw et al., 2020). Prolonged KDs acclimate multi‐organ systems to adapt to utilize lipids and ketones as the primary energy sources (Ma et al., 2018), while acute ketosis induced by exogenous ketone ingestion leaves the body remaining in a non‐keto‐adapted (keto‐naïve) state, meaning that it has not adapted to enhance the utilization of lipids and ketones (Kackley et al., 2020).

Keto‐adaptation, characterized by enhanced fat mobilization and oxidation capacity, may be crucial for optimizing the ergogenic potential of exogenous ketone supplementation (Ma & Suzuki, 2019; Shaw et al., 2020). Investigating the impact of exogenous ketone supplementation in the context of keto‐adaptation may provide valuable insights into the potential synergistic effects of these two nutritional strategies and guide the development of novel sports nutrition approaches (Ma & Suzuki, 2019). It is important to note that the current human literature on ketone supplementation and exercise performance shows mixed results, with most studies reporting neutral or negative effects on performance (Evans et al., 2022; Valenzuela et al., 2020). A recent review by Burke and colleagues (2024) in Medicine & Science in Sports & Exercise argued that ketogenic diets are not beneficial for athletic performance in most contexts (Burke & Whitfield, 2024). However, most human studies have examined acute ketone supplementation in keto‐naïve individuals, leaving the effects of chronic supplementation in keto‐adapted states relatively unexplored.

Furthermore, our previous studies have shown that a KD may reduce exhaustive exercise‐induced muscle and organ damage (Ma et al., 2018; Ma & Suzuki, 2019; Suzuki et al., 2020). Recent research has also highlighted ketones' role as signaling molecules that modulate anti‐inflammatory responses (Nelson et al., 2023; Puchalska & Crawford, 2017), potentially ameliorating exercise‐induced inflammation and subsequent tissue damage. To explore these protective effects, we will investigate markers of muscle and organ damage, such as aspartate aminotransferase (AST) and alanine aminotransferase (ALT), in response to exhaustive exercise under different ketone supplementation conditions. This approach will allow us to assess whether exogenous ketone supplementation can mitigate exercise‐induced tissue damage, possibly through its anti‐inflammatory and antioxidant properties.

Based on the above hypotheses, this study aims to investigate the effects of exogenous ketone supplementation on keto‐adapted states. While a KD may help achieve stable circulating ketone body levels of around 3 mM, evidence suggests that exogenous ketone supplementation can further elevate this concentration to higher levels (e.g., Cox et al. reported a rise to around 6 mM (Cox et al., 2016)). Ketone bodies have been shown to act not only as metabolic substrates but also as signaling molecules capable of modulating gene expression and cellular functions. For example, β‐hydroxybutyrate (BHB) can activate G protein‐coupled receptors such as GPR109A, influencing inflammatory responses and metabolic regulation (Xie et al., 2016). Ketone bodies have also been shown to modulate the activity of key metabolic sensors like AMPK and mTOR, potentially influencing cellular energy homeostasis and protein synthesis (Gómora‐García et al., 2023).

Therefore, we hypothesize that compared to a KD alone, supplementing exogenous ketones in the keto‐adapted state may further expand the ketone pool in vivo. As an adaptive response to the enlarged ketone pool, supplementation under a KD may exert additional effects on energy metabolism by upregulating the expression of genes involved in lipid and ketone utilization in multiple tissues. By combining in vitro and in vivo approaches, this study aims to provide a comprehensive understanding of the effects of exogenous ketone supplementation on skeletal muscle metabolism, multi‐tissue metabolic adaptations, and exercise performance in different nutritional states. The findings may offer valuable insights into the potential synergistic effects of KD and exogenous ketone supplementation, guiding the development of innovative nutritional strategies to enhance athletic performance.

## 2 MATERIALS AND METHODS

### 2.1 Animals, diet, and exogenous ketone body supplementation

The experimental procedures were approved and followed the Guiding Principles for the Care and Use of Animals in the Academic Research Ethical Review Committee of Waseda University (2021‐A13). Male C57BL/6J mice (8 weeks old, *n* = 24) were obtained from the Takasugi experimental Animals supply co., Ltd. and acclimated for 1 week. Mice were then randomly assigned to three groups (*n* = 8 per group): control diet (CON), ketogenic diet (KD), and KD with BHB supplementation (KD+BHB). The CON group received a standard chow diet (10% protein, 80% carbohydrate, 10% fat; 3.8 kcal/g, D19082304, Research Diets Inc., NJ, USA), while the KD and KD+BHB groups were fed a ketogenic diet (10% protein, 0% carbohydrate, 90% fat; 6.7 kcal/g, D10070801, Research Diets Inc.) for 6 weeks. The ketone supplement used was sodium β‐hydroxybutyrate (BHB‐Na, Sigma‐Aldrich, MO, USA). In the KD+BHB group, BHB‐Na was added to drinking water at a target dose of 0.063 g/day, equivalent to 10 g/day for a 60‐kg human based on body surface area conversion (Reagan‐Shaw et al., 2008). Water consumption was monitored daily to ensure consistent dosing, with actual daily BHB intake averaging 0.061 ± 0.005 g/day based on measured consumption. The CON and KD groups received drinking water with an equimolar amount of sodium chloride to control for sodium content. Mice consumed either BHB‐Na or sodium chloride for 3 weeks before sampling. Body weight and food intake were measured weekly, and blood ketone levels were monitored using the FreeStyle Precision Neo system (Abbott, TX, USA) throughout the study period.

It should be noted that baseline ketone levels in mice (0.5 mM in controls) are higher than in humans due to their higher metabolic rate and more frequent feeding–fasting cycles.

### 2.2 Incremental exercise test

Submaximal exercise capacity was evaluated using an incremental treadmill test at the 6 week of the study. Mice were familiarized with the treadmill 1 week before the test. The test began at 9 m/min for 9 min, increased to 10 m/min, and then increased by 2.5 m/min every 3 min, with a starting incline of 0° and an increase of 5° every 9 min (max incline 15°). Exhaustion was defined as the inability of the mouse to maintain running pace despite gentle encouragement with a soft brush.

### 2.3 Sampling and plasma biochemical assessment

At the end of the study, mice were sacrificed under anesthesia with isoflurane (Pfizer, Tokyo, Japan). Blood samples were collected from the abdominal aorta using heparin, and tissues (liver, epididymal fat, brown fat, and hindlimb muscle) were immediately excised and frozen in liquid nitrogen. Plasma was obtained by centrifugation (1500 × *g*, 10 min, 4°C) and stored at −80°C until analysis. Plasma glucose, nonesterified fatty acids (NEFA), triglyceride (TG), lipase, amylase, aspartate transaminase (AST), creatine kinase (CK), lactate dehydrogenase (LDH), blood urea nitrogen (BUN), uric acid (UA), creatinine, total cholesterol (T‐CHO), high‐density lipoprotein cholesterol (HDL‐CHO), low‐density‐lipoprotein cholesterol (LDL‐CHO), and albumin were measured by Koutou‐Biken Co. (Tsukuba, Japan). Blood BHB concentration and non‐fasting blood glucose were measured using the Abbott glucose and ketone meter (Abbott). Tissue glycogen was measured using the EnzyChrom™ Glycogen Assay Kit (BioAssay Systems, CA, USA) according to the manufacturer's instructions.

### 2.4 RNA isolation and real‐time PCR

Total RNA was extracted from the gastrocnemius and soleus muscle tissues (RNeasy Fibrous Mini Kit), white and brown adipose tissues (RNeasy Lipid Mini Kit), and heart and liver (RNeasy Mini Kit) (all kits from Qiagen, CA, USA). cDNA synthesis was performed using the High‐Capacity cDNA Reverse Transcription Kit (Applied Biosystem, CA, USA), and real‐time PCR was conducted using the Fast 7500 real‐time PCR system (Applied Biosystem) and Fast SYBR® Green PCR Master Mix (Applied Biosystem). The thermal profiles consisted of 10 min at 95°C for denaturation followed by 40 cycles of 95°C for 3 s and annealing at 60°C for 15 s. 18S ribosomal RNA was used as the housekeeping gene, and the ∆∆Ct method was used. All data are represented relative to their expression as fold change to the CON group.

### 2.5 Protein isolation and Western blot

Total protein was isolated from the gastrocnemius muscle and liver using T‐PER Tissue Protein Extraction Reagent (#78510, Thermo Fisher) supplemented with protease and phosphatase inhibitors (cOmplete™ Protease Inhibitor Cocktail, #4693116001, and PhosSTOP, #4906845001, Roche, Basel, Switzerland). Protein concentration was determined using the Pierce BCA protein assay (23225, Thermo Fisher). Samples were prepared with 4× Laemmli Sample Buffer (#1610747, Bio‐Rad, CA, USA), heated at 95°C for 5 min, and separated by SDS‐PAGE (Mini‐PROTEAN TGX gels, #4561096, Bio‐Rad). Primary antibodies against AMPKα (#2532), phospho‐AMPKα (#2531), mTOR (#2971), and phospho‐mTOR (#2972) (all from Cell Signaling Technology, MA, USA) were used at a 1:1000 dilution. Anti‐rabbit IgG HRP‐linked antibody (31460, Thermo Fisher) was used as the secondary antibody at a 1:5000 dilution. Protein bands were visualized using enhanced chemiluminescence reagents (SuperSignal West Pico PLUS, Thermo Fisher) and quantified using Image J software (NIH, USA).

### 2.6 Statistical analysis

Data are presented as mean ± standard error of the mean (SEM) with individual data points shown in figures. Differences among groups were analyzed using one‐way analysis of variance (ANOVA) followed by Tukey's honestly significant difference (HSD) post hoc test for all pairwise comparisons. For pre‐ and post‐exercise measurements, repeated measures ANOVA was used with group as the between‐subjects factor and time as the within‐subjects factor. Effect sizes (Cohen's *d*) and 95% confidence intervals for between‐group differences were calculated. Statistical significance was set at *p* < 0.05. Exact *p* values are reported throughout. All statistical analyses were performed using GraphPad Prism 9.0 (GraphPad Software, CA, USA).

## 3 RESULTS

### 3.1 Chronic ketone body supplementation in keto‐adapted mice further increases circulating BHB and promotes glycogen‐sparing metabolism during exercise

Table 1 shows the final blood chemistry at the end of this study. KD induced an increase in circulating BHB from 0.5 ± 0.1 mM in the CON group to 1.96 ± 0.34 mM (2.9‐fold increase compared to CON, *p* < 0.01). This was further elevated by BHB administration upon KD to 2.63 ± 0.53 mM (difference from KD: 0.67 mM, 95% CI: 0.12–1.22, *p* < 0.05; difference from CON: 2.12 mM, 95% CI: 1.57–2.67, *p* < 0.01; Cohen's *d* = 1.8 vs. KD, *d* = 3.2 vs. CON).

**TABLE 1:** Biochemical results of at the end of Study 3.

	CON	KD	KD + BHB
Albumin (mg/dL)	2.70 ± 0.32	3.04 ± 0.21	2.82 ± 0.29
BHB (mmol/L)	0.51 ± 0.12	1.96 ± 0.34**	2.63 ± 0.53** ^,^ ^#^
Glucose (mg/dL)	202 ± 23.5	182 ± 64.8	154 ± 38.7*
TG (mg/dL)	9.4 ± 4.0	25.6 ± 12.9**	19.1 ± 7.6 (*p* = 0.10 vs. CON)
NEFA (μEq/mL)	1.32 ± 0.41	2.63 ± 0.70***	2.65 ± 0.66**
T‐Cho (mg/dL)	82.9 ± 27.0	127 ± 23.6**	118 ± 23.6*
HDL‐CHO (mg/dL)	62.6 ± 22.3	105 ± 21.6**	96.9 ± 23.7*
LDL‐CHO (mg/dL)	13.5 ± 5.78	15.4 ± 4.03	12.9 ± 5.93
Creatinine (mg/dL)	0.09 ± 0.02	0.15 ± 0.05*	0.12 ± 0.05
Uric Acid (mg/dL)	1.13 ± 0.75	1.24 ± 0.80	1.16 ± 0.94
Amylase (IU/L)	1462 ± 306	1450 ± 247	1359 ± 234
Lipase (IU/L)	43.5 ± 10.5	81.9 ± 23.6**	81.9 ± 31.1*
Glycogen, Soleus Muscle (μg/g Protein)	2.14 ± 0.62	1.19 ± 0.37**	1.14 ± 0.19**
Glycogen, Liver (μg/g Protein)	3.61 ± 1.55	2.44 ± 0.79 (*p* = 0.09 vs. CON)	2.29 ± 0.82 (*p* = 0.06 vs. CON)

*Note*: Values are means ± SE; *n* = 7–8. Comparisons were made by one‐way ANOVA with Tukey's post hoc correction. Effect sizes (Cohen's *d*) for KD versus CON and KD+BHB versus CON comparisons: BHB (*d* = 3.1, *d* = 3.8), Glucose (*d* = 0.4, *d* = 1.2), TG (*d* = 1.4, *d* = 1.0), NEFA (*d* = 2.1, *d* = 2.2), Glycogen‐Soleus (*d* = 1.8, *d* = 2.3), and Glycogen‐Liver (*d* = 0.9, *d* = 1.0).

Abbreviation: BHB, β‐hydroxybutyric acid.

* *p* < 0.05.

** *p* < 0.01 versus CON.

*** *p* < 0.001 versus CON.

^#^ *p* < 0.05 versus KD.

In contrast, non‐fasting glucose level was slightly lower in the KD group (10% decrease compared to CON, not significant), while significantly lower in the KD+BHB group compared with CON (24% decrease, *p* < 0.01).

Although KD dramatically increased plasma TG (25.6 ± 12.9 mg/dL, *p* < 0.01 vs. CON), this increase was partially attenuated in the KD + BHB group (19.1 ± 7.6 mg/dL, *p* = 0.10 vs. CON). NEFA was increased in both KD (2.63 ± 0.70 μEq/mL, *p* < 0.001 vs. CON) and KD+BHB (2.65 ± 0.66 μEq/mL, *p* < 0.001 vs. CON) groups.

KD or KD+BHB significantly increased total cholesterol (127 ± 23.6 mg/dL and 118 ± 23.6 mg/dL, respectively, *p* < 0.01 and *p* < 0.05 vs. CON) and HDL‐cholesterol (105 ± 21.6 mg/dL and 96.9 ± 23.7 mg/dL, respectively, *p* < 0.01 and *p* < 0.05 vs. CON), while LDL‐cholesterol remained unaltered.

Lipase activity was also increased in both KD (81.9 ± 23.6 IU/L, *p* < 0.01 vs. CON) and KD+BHB (81.9 ± 31.1 IU/L, *p* < 0.05 vs. CON) groups.

Regarding glycogen content, soleus muscle glycogen was significantly decreased in both KD (1.19 ± 0.37 μg/g protein, *p* < 0.01) and KD+BHB (1.14 ± 0.19 μg/g protein, *p* < 0.01) groups compared to CON. Liver glycogen was lower in both KD (2.44 ± 0.79 μg/g protein, difference from CON: −1.17 μg/g, *p* = 0.09, *d* = 0.98) and KD + BHB (2.29 ± 0.82 μg/g protein, difference from CON: −1.32 μg/g, *p* = 0.06, *d* = 1.04) groups compared to CON (3.61 ± 1.55 μg/g protein).

Other parameters, albumin, creatinine, uric acid, and amylase were not altered neither in KD nor KD+BHB groups (*p* > 0.05 vs. CON).

An incremental exercise test was conducted at Week 6, and Table 2 shows the biochemical results before and after the incremental exercise test. Neither KD nor KD+BHB intervention significantly alters the maximal exercise time. Glucose and BHB in the blood are measured before and immediately after exercise.

**TABLE 2:** Exercise time and biochemical results before and after exercise of Study 3.

	CON	KD	KD + BHB
Maximal exercise time (minutes)	31 ± 4	27 ± 5	32 ± 5
Pre‐exercise glucose	172 ± 42	159 ± 40	130 ± 40 (*p* = 0.09 vs. CON)
Post‐exercise glucose	169 ± 37	195 ± 72	188 ± 40
Δglucose (mg/dL)	−2 ± 37	46 ± 56 (*p* = 0.09 vs. CON)	51 ± 33 (*p* = 0.06 vs. CON)
Pre‐exercise BHB	0.5 ± 0.1	2.0 ± 0.3**	2.6 ± 0.5***,#
Post‐exercise BHB	0.8 ± 0.2	1.3 ± 0.3*	1.5 ± 0.2**
ΔBHB (μM)	0.3 ± 0.3	−0.6 ± 0.2**	−1.2 ± 0.4** ^,^ ^#^

*Note*: Δglucose and ΔBHB means the concentration of these indicators post‐exercise subtract those before exercise. Values are means ± SE; *n* = 7–8. Comparisons were made by one‐way ANOVA with Tukey's post hoc correction. Repeated measures ANOVA showed a significant group × time interaction for BHB (*F* (2, 21) = 18.3, *p* < 0.001) and glucose (*F* (2, 21) = 4.2, *p* = 0.029). Effect sizes for ΔBHB: KD versus CON (*d* = 2.8), KD+BHB versus CON (*d* = 3.5), KD+BHB versus KD (*d* = 1.4).

Abbreviation: BHB, β‐hydroxybutyric acid.

* *p* < 0.05.

** *p* < 0.01 versus CON.

*** *p* < 0.001 versus CON.

^#^ *p* < 0.05 versus KD.

During exercise, the KD and KD+BHB groups showed significant decreases in blood BHB levels (ΔBHB = ‐0.6 ± 0.2 μM and −1.2 ± 0.4 μM, respectively), while the CON group showed a slight increase (ΔBHB = 0.3 ± 0.3 μM). This suggests increased ketone utilization during exercise in keto‐adapted states.

Blood glucose increased during exercise in KD (Δglucose = 46 ± 56 mg/dL, difference from CON: 48 mg/dL, *p* = 0.09, *d* = 0.94) and KD+BHB (Δglucose = 51 ± 33 mg/dL, difference from CON: 53 mg/dL, *p* = 0.06, *d* = 1.12) groups, while it remained stable in CON (Δglucose = −2 ± 37 mg/dL). The above results indicate a potential glucose‐sparing effect in keto‐adapted mice during exercise.

### 3.2 Chronic ketone body supplementation further altered genes expression profiles in heart, liver, adipose tissue, and skeletal muscle tissue

**FIGURE 1:**
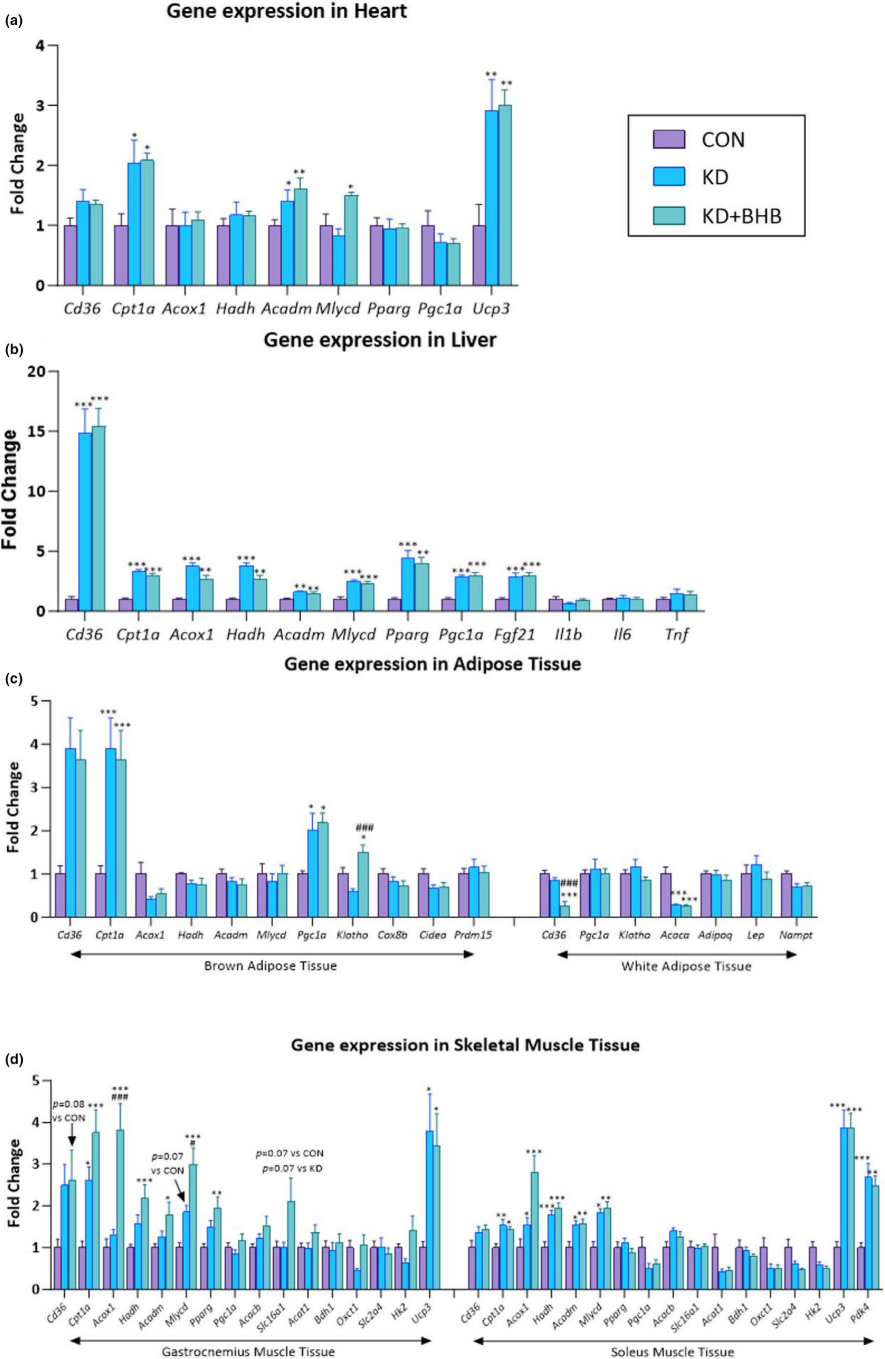
Effects of chronic ketogenic diet (KD) and BHB administration on gene expression in multiple tissues. mRNA expression levels in (a) heart, (b) liver, (c) adipose tissues (brown and white), and (d) skeletal muscle tissues (gastrocnemius and soleus). Purple bars: Control diet (CON); light blue bars: Ketogenic diet (KD); dark blue bars: Ketogenic diet with BHB supplementation (KD+BHB). Values are expressed as mean ± SE (*n* = 7–8). Statistical analysis: One‐way ANOVA with Dunnett's post hoc correction. *p < 0.05, **p < 0.01, ***p < 0.001 compared with CON; ^#^ *p* < 0.05, ^##^ *p* < 0.01, ^###^ *p* < 0.001 compared with KD. For panel d, additional *p* values are noted for specific comparisons. Individual data points are shown for each measurement. Sample sizes: *n* = 8 for CON, *n* = 7–8 for KD and KD+BHB due to technical issues with RNA extraction from some samples.

We measured several gene sets related to lipid metabolism, glucose metabolism, and energy regulation. Gene expression profiles were evaluated in the heart, liver, gastrocnemius, and soleus muscle tissue (Figure 1).

In the heart, *Cpt1a* (fatty acid translocase), *Acadm* (fatty acid oxidation), and *Ucp3* (uncoupling protein, energy metabolism) were upregulated by both KD and KD+BHB (*p* < 0.05 for all), while *Cd36* (fatty acid translocase), *Hadh* (fatty acid oxidation), *Pparg* (lipid metabolism and adipocyte differentiation), and *Pgc1a* (mitochondrial biogenesis and energy metabolism) remained unchanged. *Mlycd* (fatty acid synthesis) was significantly increased only in the KD+BHB group (*p* < 0.05) (Figure 1a).

In the liver, *Cd36*, *Cpt1a*, *Acox1* (peroxisomal fatty acid oxidation), *Hadh*, *Acadm*, *Mlycd*, *Pparg*, *Pgc1a*, and *Fgf21* (metabolic regulation) were significantly increased in both KD and KD+BHB groups (*p* < 0.05 for all, Figure 1b). Unlike acute administration, chronic BHB supplementation did not induce upregulation of genes encoding *Tnf*, *Il1b*, or *Il6* (Figure 1b).

In brown adipose tissue, KD and KD+BHB significantly enhanced *Cd36*, *Cpt1a*, and *Pgc1a* expression (*p* < 0.05 for all). Only KD+BHB induced a significant increase in *Klotho* expression (*p* < 0.05 compared to both CON and KD groups). *Klotho* encodes the receptor for FGF21 and is involved in metabolic regulation. Among the genes evaluated in brown adipose tissue, *Acox1*, *Hadh*, *Acadm*, *Mlycd*, and *Cox8b* (mitochondrial function and thermogenesis), *Cidea* (thermogenesis), and *Prdm15* (brown adipocyte differentiation and thermogenesis) remained unchanged (Figure 1c).

In white adipose tissue, *Acaca* (fatty acid synthesis) was decreased in both KD and KD+BHB groups (*p* < 0.05 for both comparisons), while *Cd36* was significantly decreased only in the KD+BHB group (*p* < 0.05). Among the genes evaluated in white adipose tissue, *Pgc1a*, *Klotho*, *Adipoq* (adiponectin, insulin sensitivity and fatty acid oxidation), *Lep* (leptin, energy balance and appetite regulation), and *Nampt* (visfatin, nicotinamide phosphoribosyltransferase, NAD+ biosynthesis) remain unchanged (Figure 1c).

In gastrocnemius muscle, KD or KD+BHB significantly increased the expression of *Cpt1a* and *Ucp3* (*p* < 0.05). Additionally, KD+BHB increased the expression of *Acox1*, *Hadh*, *Acadm*, *Mlycd*, and *Pparg* (*p* < 0.05). Cd36, *Pgc1a*, *Acacb*, *Slc16a1* (monocarboxylate transporter 1, lactate and ketone body transport), *Bdh1* (ketone body utilization), *Oxct1* (ketone body utilization), *Slc2a4* (glucose transporter 4, glucose uptake), and *Hk2* (glycolysis) were not changed by either intervention (Figure 1d).

In soleus muscle, both KD and KD+BHB increased the expression of *Cpt1a*, *Acox1*, *Hadh*, *Acadm*, *Mlycd*, *Ucp3*, and *Pdk4* (glucose/lipid metabolism regulator). *Cd36*, *Pparg*, *Pgc1a*, *Acacb*, *Slc16a1*, *Acat1*, *Bdh1*, *Oxct1*, *Slc2a4*, and *Hk2* remained unchanged. For *Acox1*, an increasing trend is found between KD+BHB and KD (Figure 1d).

### 3.3 Chronic ketone body supplementation activates AMPK but not mTOR in liver and gastrocnemius muscle

AMPK and mTOR are key regulators of cellular energy metabolism and growth, respectively. AMPK is activated under energy‐deficient conditions, such as during exercise or fasting, and promotes catabolic pathways to generate ATP, while inhibiting anabolic pathways. In contrast, mTOR is activated under energy‐sufficient conditions and promotes anabolic processes, such as protein synthesis and cell growth. Given the potential impact of ketone body supplementation on energy metabolism, we investigated the phosphorylation status of AMPK and mTOR in the liver and gastrocnemius muscle.

**FIGURE 2:**
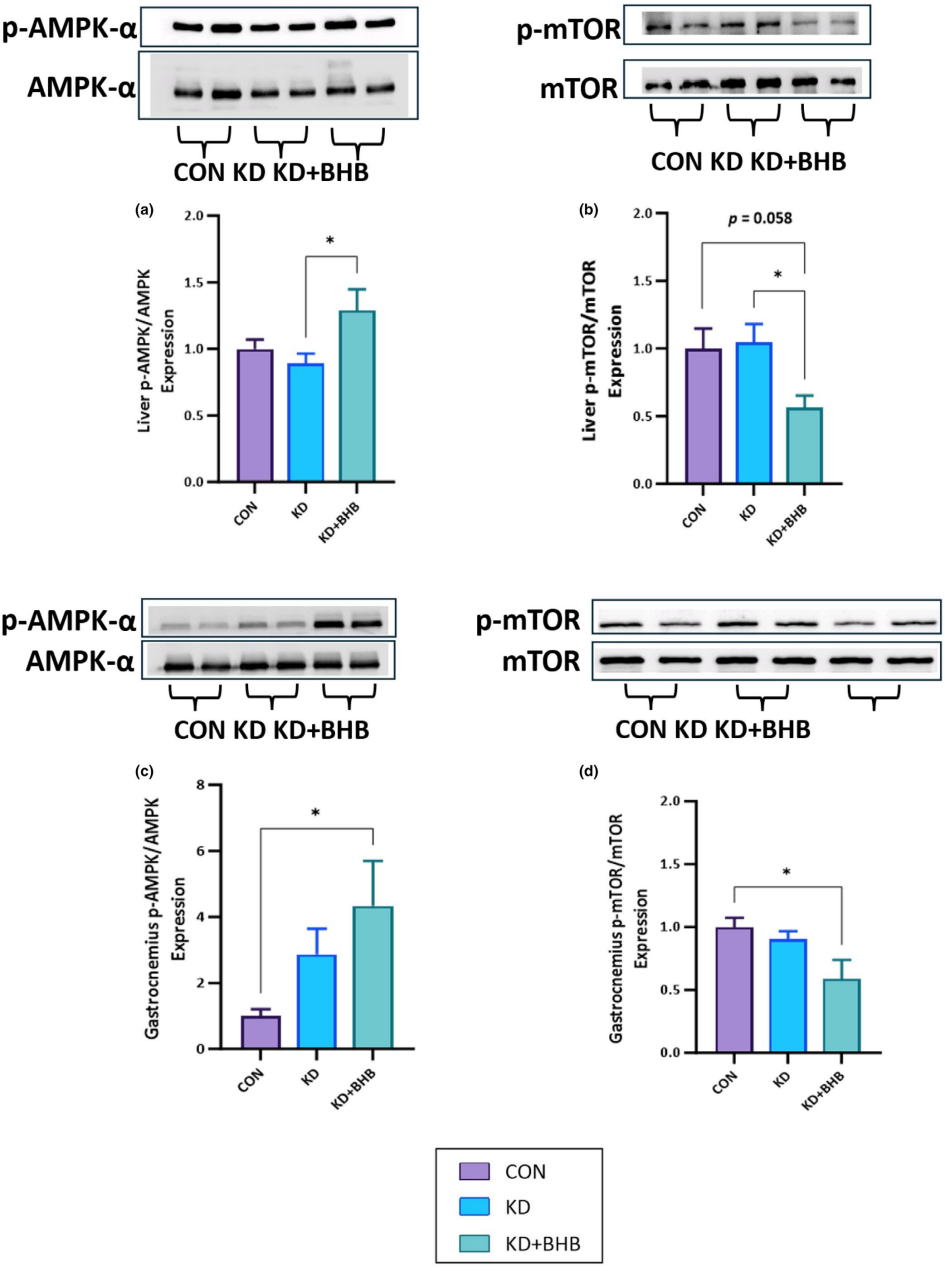
Effects of chronic ketogenic diet (KD) and BHB administration on AMPK and mTOR phosphorylation in liver and gastrocnemius muscle. Western blot analysis and quantification of: (a) p‐AMPK‐α/AMPK‐α ratio in liver (b) p‐mTOR/mTOR ratio in liver (c) p‐AMPK‐α/AMPK‐α ratio in gastrocnemius muscle (d) p‐mTOR/mTOR ratio in gastrocnemius muscle. Representative Western blot images are shown above each graph for p‐AMPK‐α, total AMPK‐α, p‐mTOR, and total mTOR. Purple bars: Control diet (CON); light blue bars: Ketogenic diet (KD); dark blue bars: Ketogenic diet with BHB supplementation (KD+BHB). Values are expressed as mean ± SEM (*n* = 3–6). Statistical analysis: One‐way ANOVA with Dunnett's post hoc correction. *p < 0.05. Sample sizes: *n* = 6 per group for Western blot analysis due to limited tissue availability and technical constraints. Despite the smaller sample size, significant differences were detected with large effect sizes (*d* > 1.5 for significant comparisons).

In the liver, KD+BHB significantly increased phosphorylated AMPK and decreased phosphorylated mTOR compared to KD (Figure 2a, *p* = 0.058 between CON and KD+BHB for mTOR).

Similarly, in gastrocnemius muscle, KD+BHB significantly increased phosphorylated AMPK while decreasing phosphorylated mTOR, compared to CON (Figure 2b, *p* = 0.058 between CON and KD+BHB for mTOR).

## 4 DISCUSSION

The effects of ketone supplementation on exercise metabolism and performance remain controversial, with human studies showing predominantly neutral or negative results (Margolis & O'Fallon, 2020; Puchalska & Crawford, 2021). However, most investigations have examined acute ketone supplementation in keto‐naïve individuals. Our study addressed this gap by examining chronic ketone supplementation in keto‐adapted mice, revealing unique metabolic adaptations rather than performance enhancement per se.

Our study addressed these conflicting findings by examining ketone supplementation effects through a novel lens: the subject's metabolic state, particularly keto‐adaptation (Ma et al., 2018; Ma & Suzuki, 2019). While ketone bodies show favorable metabolic effects in certain contexts (Clark et al., 2021), their impact on exercise capacity has been inconsistent when administered acutely (Shaw et al., 2020).

Our findings on chronic ketone supplementation in keto‐adapted mice revealed a notable glycogen‐thrifty effect during exercise. Despite significantly lower resting muscle glycogen content in both KD and KD+BHB groups, exercise capacity was maintained comparable to controls. This glycogen conservation effect is supported by several observations: (1) upregulation of fatty acid oxidation genes across multiple tissues; (2) increased AMPK phosphorylation with decreased mTOR phosphorylation, creating a metabolic environment favoring fat utilization; (3) greater decrease in blood BHB levels during exercise in the KD+BHB group (ΔBHB = −1.2 ± 0.4 μM vs. −0.6 ± 0.2 μM in KD), indicating enhanced ketone utilization; and (4) maintenance of blood glucose during exercise in keto‐adapted states versus the decrease observed in controls. Together, these results highlight a significant metabolic adaptation potentially beneficial for endurance performance.

The term “glycogen‐thrifty” requires clarification. We do not suggest that glycogen stores are conserved in absolute terms—both KD groups had significantly lower resting glycogen. Rather, this term describes the ability to maintain exercise capacity despite these reduced stores, suggesting more efficient utilization of limited glycogen reserves combined with enhanced reliance on alternative fuels. This concept differs from traditional “glycogen sparing,” which implies preservation of glycogen content. Our findings align with observations in glycogen storage diseases where ketogenic diets can maintain function despite impaired glycogen metabolism (Løkken et al., 2020, 2022).

The glycogen‐thrifty phenomenon we observed challenges the traditional carbohydrate‐loading paradigm in exercise nutrition. While glycogen depletion is conventionally considered a primary limiting factor in endurance exercise, our results suggest keto‐adapted mice with ketone supplementation maintain exercise capacity despite reduced glycogen reserves. This metabolic flexibility represents a fundamental shift in understanding fuel utilization during exercise, aligning with Volek et al.'s findings (Volek et al., 2016) in keto‐adapted ultra‐endurance athletes who maintained performance despite lower glycogen utilization.

Regarding the energetic sufficiency of circulating ketones to sustain exercise, our data suggest that the circulating ketone pool alone cannot account for the maintained exercise capacity. With blood BHB levels of 2.63 mM in the KD+BHB group, the total circulating ketone pool would provide approximately 0.5–1.0 mmol/kg body weight. Given that BHB oxidation yields approximately 21.5 mol ATP per mol (compared to 32 mol ATP/mol glucose), this circulating pool could theoretically sustain high‐intensity exercise for only 3–5 min, not the 30+ min observed in our exhaustive exercise test. This calculation reveals that circulating ketones serve as a supplementary rather than primary fuel source. The maintained exercise capacity despite lower glycogen must therefore result from the coordinated utilization of multiple substrates—enhanced fatty acid oxidation (evidenced by elevated NEFA and upregulated fat oxidation genes), efficient ketone utilization (demonstrated by the 1.2 mM decrease in BHB during exercise), and strategic preservation of the limited glucose/glycogen reserves (shown by the paradoxical glucose increase during exercise in KD groups). This multi‐substrate metabolic flexibility, rather than ketone bodies alone replacing glucose, underlies the glycogen‐thrifty phenomenon.

The apparent paradox of sustained NEFA elevation despite BHB supplementation deserves careful consideration. In acute exogenous ketosis, BHB rapidly activates the HCAR2 (GPR109A) receptor on adipocytes, triggering Gi‐protein signaling that inhibits hormone‐sensitive lipase and suppresses lipolysis, typically reducing NEFA by 30%–50% within hours (Zhao et al., 2023). However, our model fundamentally differs from acute supplementation studies in three critical aspects:

First, the chronic nature of our supplementation (3 weeks) likely induces HCAR2 receptor desensitization or downregulation. Similar to beta‐adrenergic receptor desensitization during chronic catecholamine exposure, sustained HCAR2 activation may lead to receptor internalization and reduced coupling efficiency. This would explain why the anti‐lipolytic effect is attenuated, allowing NEFA levels to remain elevated despite high BHB concentrations.

Second, the metabolic milieu of keto‐adaptation creates a fundamentally different hormonal environment. In our keto‐adapted mice, the metabolic profile suggests maintained insulin suppression typical of ketogenic diets. While we did not measure insulin directly, the sustained elevation of NEFA (2.65 ± 0.66 μEq/mL) and reduced blood glucose (154 ± 38.7 mg/dL in KD+BHB) are consistent with low insulin signaling, as elevated insulin would typically suppress lipolysis and reduce NEFA levels. This hormonal profile strongly favors lipolysis through multiple pathways that can override HCAR2‐mediated suppression. The phosphorylation of hormone‐sensitive lipase by PKA remains elevated due to low insulin signaling, while ATGL expression is enhanced by PPARα activation (which we observed through increased Pparg expression). These pro‐lipolytic signals may compensate for any HCAR2‐mediated inhibition.

Third, and perhaps most importantly, the enhanced fatty acid oxidation capacity in our KD+BHB mice creates a “pull” effect on adipose tissue lipolysis. The upregulation of *Cpt1a*, *Acadm*, and other fat oxidation genes across multiple tissues increases fatty acid clearance and turnover. This creates a metabolic demand that stimulates continued lipolysis despite the presence of ketones. The liver, in particular, shows dramatic upregulation of fat oxidation machinery, which may maintain hepatic ATP levels necessary for continued ketogenesis, creating a feed‐forward cycle.

This contrasts starkly with acute exogenous ketosis in keto‐naïve subjects, where insulin is stimulated (often increasing 2–3 fold), HCAR2 activation is maximal, and tissues lack the enzymatic machinery for efficient fat oxidation. In that context, NEFA suppression is pronounced and fat oxidation is actually reduced. Our findings suggest that chronic keto‐adaptation fundamentally rewires the metabolic response to exogenous ketones, transforming BHB from an anti‐lipolytic signal to a co‐substrate that enhances overall metabolic flexibility.

Chronic exogenous ketone supplementation effectively expanded the ketone body pool beyond what the ketogenic diet alone achieved (2.63 ± 0.53 mM vs. 1.96 ± 0.34 mM), providing additional metabolic benefits in terms of glycogen conservation. Cox et al. (Cox et al., 2016) demonstrated that elevated ketone levels can decrease glycolysis during exercise in humans, which aligns with our findings of maintained exercise capacity despite lower glycogen content, particularly important during prolonged exercise where glycogen depletion typically limits performance.

Our model represents a unique metabolic state—chronic exogenous ketone supplementation superimposed on endogenous ketosis from keto‐adaptation. This differs fundamentally from acute exogenous ketone supplementation in keto‐naïve subjects. In the latter, insulin is typically stimulated and NEFA suppressed via HCAR2 receptor activation (Stubbs et al., 2017). In contrast, our keto‐adapted mice maintained the metabolic milieu characteristic of nutritional ketosis (low insulin, high NEFA) despite exogenous BHB supplementation. This may reflect HCAR2 receptor desensitization or compensatory mechanisms that preserve lipolytic capacity during chronic exposure. The sustained elevation of NEFA in our KD+BHB group, despite BHB's known anti‐lipolytic effects, suggests that chronic adaptation modifies the acute metabolic responses to exogenous ketones.

The upregulation of fatty acid oxidation genes (e.g., *Cpt1a* and *Acadm*) across multiple tissues provides a molecular basis for the observed glycogen‐thrifty effect. By enhancing fat oxidation capacity, these adaptations may reduce reliance on glycogen during exercise, preserving limited glycogen stores. The KD+BHB group often showed more pronounced gene expression changes than KD alone, suggesting ketone supplementation upon keto‐adaptation may further enhance these beneficial adaptations.

The paradox of maintained exercise capacity despite reduced glycogen content represents a significant finding. This glycogen‐thrifty effect may be explained by enhanced capacity for fat and ketone utilization, increased metabolic efficiency, and altered substrate competition.

Previous ex vivo studies have shown that 4 mmol/L BHB supplementation significantly enhanced glycogen repletion in mouse epitrochlearis muscle during 120 min of post‐exercise recovery (Takahashi et al., 2019). Although our study demonstrated a glycogen‐lowering effect at rest in the gastrocnemius muscle, the maintained exercise capacity suggests that the glycogen‐thrifty effect during exercise may compensate for lower resting glycogen levels. This discrepancy highlights the need for further investigation into glycogen‐sustaining effects across different muscle fiber types and how chronic BHB supplementation upon keto‐adapted state helps to sustain exercise tolerance despite reduced glycogen reserves.

The opposing changes in AMPK (increased) and mTOR (decreased) phosphorylation in KD+BHB mice create a metabolic environment favoring catabolism over anabolism. While this state resembles caloric restriction, our mice maintained normal food intake after the initial adaptation period (Table S1). This suggests that ketone supplementation in keto‐adapted states might confer some metabolic benefits of caloric restriction without reduced energy intake, supporting the “ketone signaling” hypothesis. Furthermore, recent work by Crawford's group has shown that ketones can be incorporated into newly synthesized lipids, particularly in cardiac tissue (Queathem, Moazzami, et al., 2025; Queathem, Stagg, et al., 2025), which may explain the upregulation of fatty acid synthesis genes (e.g., *Mlycd*) we observed despite the overall catabolic state.

Notably, while our results suggest a calorie restriction‐like state, mice were not calorically restricted, implying ketone supplementation in keto‐adapted states might confer some benefits of calorie restriction without reduced food intake. This aligns with the “ketone signaling” hypothesis, suggesting ketone bodies act as signaling molecules that modulate cellular processes beyond their role as metabolic substrates.

Our findings have significant implications for exercise nutrition and performance. By maintaining exercise capacity despite lower glycogen reserves, ketone supplementation in keto‐adapted states may provide metabolic advantages during prolonged exercise where glycogen depletion typically limits performance. This effect may be particularly relevant for ultra‐endurance events requiring metabolic flexibility and efficient substrate utilization. The glycogen‐thrifty effect may also have implications beyond exercise performance, potentially benefiting conditions characterized by impaired glucose metabolism or glycogen storage.

Several limitations should be acknowledged. First, we did not include a keto‐naïve + BHB group, which would have allowed direct comparison of BHB effects in adapted versus non‐adapted states. Second, without respiratory exchange ratio (RER) measurements, we cannot definitively quantify substrate utilization during exercise; our conclusions about enhanced fat oxidation rely on gene expression and circulating metabolite data. Third, the exhaustive exercise protocol used may not translate to typical human endurance performance, and the lack of training stimulus limits applicability to athletic populations. Fourth, our BHB dose (equivalent to ~10 g/day in humans) is lower than many acute supplementation studies (typically 0.5 g/kg), though it aligns with chronic supplementation protocols. Finally, metabolic differences between mice and humans, including higher basal metabolic rates and ketone levels in mice, may limit translational relevance. Future studies should address these limitations with dose–response designs, direct metabolic measurements, and inclusion of appropriate control groups.

## 5 CONCLUSION

In summary, chronic ketone body supplementation in keto‐adapted mice leads to significant metabolic adaptations, including a notable glycogen‐thrifty effect during exercise. Despite lower resting glycogen content, exercise capacity was maintained, suggesting enhanced metabolic flexibility and efficient substrate utilization. These adaptations appear more pronounced than with the ketogenic diet alone, suggesting a potential synergistic effect of combining the ketogenic diet with exogenous ketone supplementation. The molecular mechanisms underlying these adaptations collectively create a metabolic environment favoring glycogen conservation during exercise.

## AUTHOR CONTRIBUTIONS

S. M. and Y. K. conceived and designed the research. S. M., C. W., and Y. T. performed the experiments and analyzed the data. S. M. interpreted the results of the experiments, prepared the figures, and drafted the manuscript; K. S. and T. H. edited and revised the manuscript; all authors approved the final version of the manuscript.

## FUNDING INFORMATION

Part of this study is funded by Japan Society for the Promotion of Science, to Sihui Ma: 20F20111 and 23K10910.

## CONFLICT OF INTEREST STATEMENT

The author declared no potential conflicts of interest with respect to the research, authorship, and/or publication of this article.

## ETHICS STATEMENT

All animal experimental procedures were approved by the Academic Research Ethical Review Committee of Waseda University (approval number: 2021‐A13) and were conducted in accordance with the Guiding Principles for the Care and Use of Animals.

## Supporting information


Appendix S1.

## Data Availability

All data supporting the findings of this study are available from the corresponding author upon reasonable request. Raw data will be deposited in the Figshare repository upon manuscript acceptance.
