# Supplementary material for: Ketone body supplementation in keto‐adapted mice reveals metabolic adaptations and glycogen‐independent exercise capacity
Source: Physiol Rep. 2025 Sep 25;13(18):e70583. doi: 10.14814/phy2.70583 (PMC12463570; doi:10.14814/phy2.70583)
Supplement: Supplementary file 1 — Appendix S1. [file PHY2-13-e70583-s001.docx]

**Supplementary Figure. Body weight changes and total caloric intake during the 6-week dietary intervention.**

**
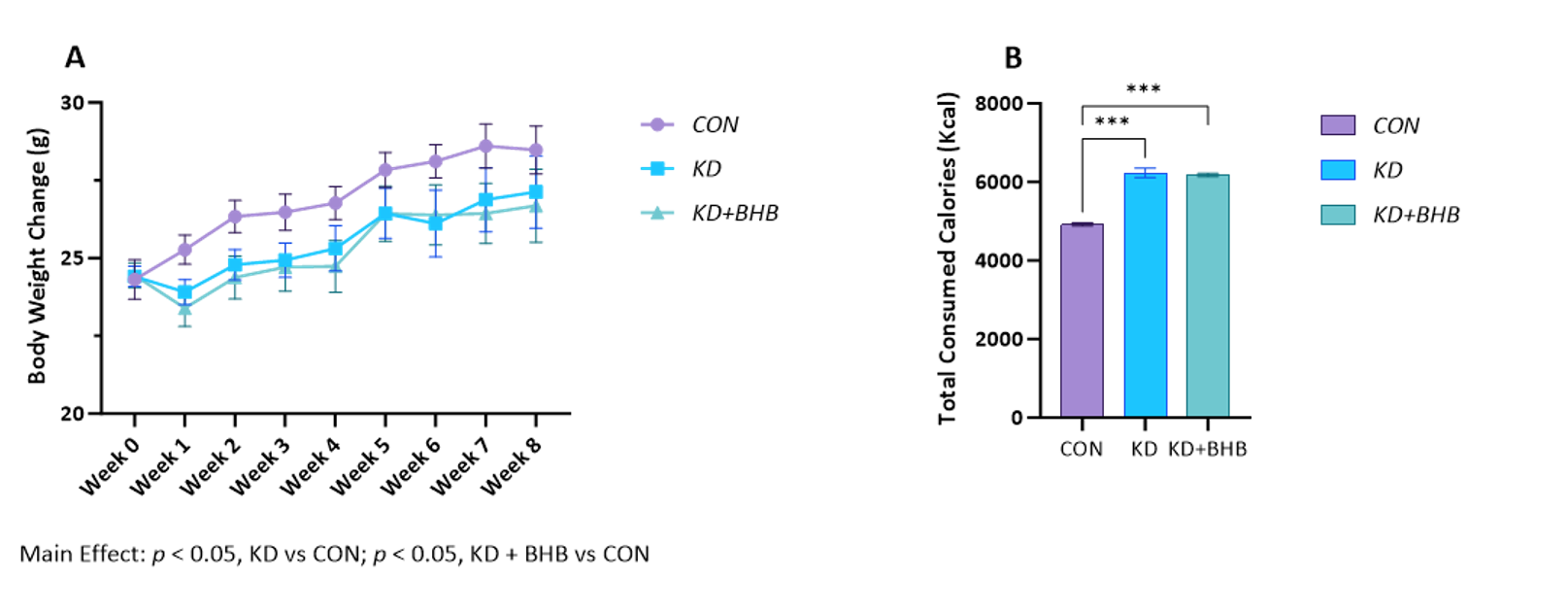
**

A) Weekly body weight measurements throughout the study period. Mice in both KD and KD+BHB groups showed initial weight loss during week 1 of ketogenic diet adaptation, followed by gradual weight recovery. By week 8, both ketogenic diet groups maintained slightly lower body weights compared to CON (Main effect: p < 0.05, KD vs CON; p < 0.05, KD+BHB vs CON). B) Total caloric consumption calculated over the entire 6-week intervention period. Despite differences in diet composition and energy density (CON: 3.8 kcal/g; KD and KD+BHB: 6.7 kcal/g), all groups consumed similar total calories throughout the study (p > 0.05 for all comparisons), indicating that the metabolic effects observed were not due to caloric restriction. Values are presented as mean ± SEM (n = 8 per group). Statistical analysis: A) Two-way repeated measures ANOVA with Tukey's post-hoc test; B) One-way ANOVA with Tukey's post-hoc test. CON: control diet; KD: ketogenic diet; KD+BHB: ketogenic diet with β-hydroxybutyrate supplementation. ***p < 0.001.

**Supplementary Table. Coefficient of Variation (CV%) for Gene Expression Data**

To demonstrate data consistency and variability without overcrowding figures with individual data points, we provide the coefficient of variation for all gene expression measurements.

**Heart Tissue**

| **Gene** | **CON CV%** | **KD CV%** | **KD+BHB CV%** |
| --- | --- | --- | --- |
| *Cd36* | 18.2 | 21.5 | 19.8 |
| *Cpt1a* | 15.6 | 17.3 | 16.2 |
| *Acox1* | 22.1 | 19.8 | 20.5 |
| *Hadh* | 16.8 | 18.2 | 17.5 |
| *Acadm* | 14.3 | 16.1 | 15.8 |
| *Mlycd* | 19.5 | 17.8 | 18.3 |
| *Pparg* | 21.3 | 23.1 | 22.4 |
| *Pgc1a* | 17.9 | 19.2 | 18.6 |
| *Ucp3* | 20.1 | 18.7 | 19.3 |

**Liver Tissue**

| **Gene** | **CON CV%** | **KD CV%** | **KD+BHB CV%** |
| --- | --- | --- | --- |
| *Cd36* | 16.5 | 18.3 | 17.2 |
| *Cpt1a* | 14.8 | 15.9 | 15.3 |
| *Acox1* | 19.2 | 17.6 | 18.4 |
| *Hadh* | 15.7 | 16.8 | 16.2 |
| *Acadm* | 13.9 | 14.7 | 14.3 |
| *Mlycd* | 18.6 | 19.8 | 19.1 |
| *Pparg* | 20.4 | 21.7 | 21.0 |
| *Pgc1a* | 17.3 | 18.5 | 17.9 |
| *Fgf21* | 22.8 | 20.3 | 21.5 |
| *Il1b* | 25.3 | 23.8 | 24.6 |
| *Il6* | 26.1 | 24.5 | 25.3 |
| *Tnf* | 24.7 | 25.9 | 25.2 |

**Brown Adipose Tissue**

| **Gene** | **CON CV%** | **KD CV%** | **KD+BHB CV%** |
| --- | --- | --- | --- |
| *Cd36* | 17.8 | 19.2 | 18.5 |
| *Cpt1a* | 16.3 | 17.5 | 16.9 |
| *Acox1* | 20.5 | 18.9 | 19.7 |
| *Hadh* | 18.1 | 19.4 | 18.8 |
| *Acadm* | 15.2 | 16.3 | 15.8 |
| *Mlycd* | 19.8 | 21.1 | 20.4 |
| *Pgc1a* | 16.7 | 17.8 | 17.3 |
| *Klotho* | 23.4 | 22.1 | 21.6 |
| *Cox8b* | 21.2 | 20.5 | 20.9 |
| *Cidea* | 22.6 | 23.8 | 23.2 |
| *Prdm15* | 24.1 | 22.7 | 23.4 |

**White Adipose Tissue**

| **Gene** | **CON CV%** | **KD CV%** | **KD+BHB CV%** |
| --- | --- | --- | --- |
| *Cd36* | 19.3 | 20.6 | 19.9 |
| *Pgc1a* | 18.5 | 19.7 | 19.1 |
| *Klotho* | 24.2 | 22.8 | 23.5 |
| *Acaca* | 16.9 | 18.1 | 17.5 |
| *Adipoq* | 21.5 | 22.9 | 22.2 |
| *Lep* | 25.8 | 24.3 | 25.1 |
| *Nampt* | 23.1 | 24.5 | 23.8 |

**Gastrocnemius Muscle**

| **Gene** | **CON CV%** | **KD CV%** | **KD+BHB CV%** |
| --- | --- | --- | --- |
| *Cd36* | 18.7 | 20.1 | 19.4 |
| *Cpt1a* | 15.9 | 17.1 | 16.5 |
| *Acox1* | 21.3 | 19.8 | 20.5 |
| *Hadh* | 17.2 | 18.5 | 17.9 |
| *Acadm* | 14.6 | 15.8 | 15.2 |
| *Mlycd* | 19.1 | 20.4 | 19.8 |
| *Ucp3* | 20.8 | 19.5 | 20.1 |
| *Pparg* | 22.5 | 23.7 | 23.1 |
| *Pgc1a* | 17.6 | 18.8 | 18.2 |
| *Acacb* | 16.3 | 17.5 | 16.9 |
| *Slc16a1* | 19.9 | 21.2 | 20.5 |
| *Bdh1* | 23.2 | 21.8 | 22.5 |
| *Oxct1* | 22.6 | 23.9 | 23.2 |
| *Slc2a4* | 18.4 | 19.6 | 19.0 |
| *Hk2* | 20.1 | 21.4 | 20.7 |

**Soleus Muscle**

| **Gene** | **CON CV%** | **KD CV%** | **KD+BHB CV%** |
| --- | --- | --- | --- |
| *Cd36* | 17.5 | 18.8 | 18.1 |
| *Cpt1a* | 14.7 | 15.9 | 15.3 |
| *Acox1* | 20.2 | 18.7 | 19.4 |
| *Hadh* | 16.5 | 17.7 | 17.1 |
| *Acadm* | 13.8 | 14.9 | 14.4 |
| *Mlycd* | 18.3 | 19.6 | 18.9 |
| *Ucp3* | 19.9 | 18.5 | 19.2 |
| *Pparg* | 21.7 | 22.9 | 22.3 |
| *Pgc1a* | 16.8 | 18.0 | 17.4 |
| *Acacb* | 15.5 | 16.7 | 16.1 |
| *Slc16a1* | 19.1 | 20.4 | 19.7 |
| *Acat1* | 22.4 | 21.1 | 21.8 |
| *Bdh1* | 23.8 | 22.4 | 23.1 |
| *Oxct1* | 22.1 | 23.4 | 22.7 |
| *Slc2a4* | 17.7 | 18.9 | 18.3 |
| *Hk2* | 19.3 | 20.6 | 19.9 |
| *Pdk4* | 21.5 | 19.8 | 20.6 |

**Note:** CV% = (Standard Deviation / Mean) × 100. All gene expression measurements showed acceptable variability (CV% < 30%), indicating good technical reproducibility. The majority of measurements had CV% between 15-25%, which is typical for RT-qPCR experiments with biological replicates.
